# PNPLA3 downregulation exacerbates the fibrotic response in human hepatic stellate cells

**DOI:** 10.1371/journal.pone.0260721

**Published:** 2021-12-08

**Authors:** Brian Rady, Takahiro Nishio, Debanjan Dhar, Xiao Liu, Mark Erion, Tatiana Kisseleva, David A. Brenner, Alessandro Pocai

**Affiliations:** 1 Cardiovascular & Metabolism, Janssen Pharmaceuticals, Spring House, PA, United States of America; 2 Department of Medicine, University of California San Diego, La Jolla, CA, United States of America; University of Navarra School of Medicine and Center for Applied Medical Research (CIMA), SPAIN

## Abstract

Non-alcoholic steatohepatitis (NASH) results, in part, from the interaction of metabolic derangements with predisposing genetic variants, leading to liver-related complications and mortality. The strongest genetic determinant is a highly prevalent missense variant in patatin-like phospholipase domain-containing protein 3 (PNPLA3 p.I148M). In human liver hepatocytes PNPLA3 localizes to the surface of lipid droplets where the mutant form is believed to enhance lipid accumulation and release of pro-inflammatory cytokines. Less is known about the role of PNPLA3 in hepatic stellate cells (HSCs). Here we characterized HSC obtained from patients carrying the wild type (n = 8 C/C) and the heterozygous (n = 6, C/G) or homozygous (n = 6, G/G) PNPLA3 I148M and investigated the effect of genotype and PNPLA3 downregulation on baseline and TGF-β-stimulated fibrotic gene expression. HSCs from all genotypes showed comparable baseline levels of PNPLA3 and expression of the fibrotic genes α-SMA, COL1A1, TIMP1 and SMAD7. Treatment with TGF-β increased PNPLA3 expression in all 3 genotypes (~2-fold) and resulted in similar stimulation of the expression of several fibrogenic genes. In primary human HSCs carrying wild-type (WT) PNPLA3, siRNA treatment reduced PNPLA3 mRNA by 79% resulting in increased expression of α-SMA, Col1a1, TIMP1, and SMAD7 in cells stimulated with TGF-β. Similarly, knock-down of PNPLA3 in HSCs carrying either C/G or G/G genotypes resulted in potentiation of TGF-β induced expression of fibrotic genes. Knockdown of PNPLA3 did not impact fibrotic gene expression in the absence of TGF-β treatment. Together, these data indicate that the presence of the I148M PNPLA3 mutation in HSC has no effect on baseline activation and that downregulation of PNPLA3 exacerbates the fibrotic response irrespective of the genotype.

## Introduction

With the increased prevalence of obesity and insulin resistance worldwide, non-alcoholic fatty liver disease (NAFLD) is becoming the leading cause of chronic liver disease and liver transplantation. Approximately 20% of NAFLD patients develop nonalcoholic steatohepatitis (NASH) characterized by lobular inflammation, hepatocyte ballooning, and fibrosis [1]. The progression of NASH has a strong genetic component, the most robust of which is a single nucleotide polymorphism (SNP) (rs738409 C->G; p.I148M) in the patatin-like phospholipase domain-containing 3 (PNPLA3) gene. Romeo et al. first reported that the rs738409 non-synonymous SNP in PNPLA3 was significantly associated with liver steatosis [2]. This finding has since been robustly replicated in different populations across the full spectrum of NAFLD, including the identification of associations with NASH histological severity and hepatic fibrosis, with confirmation of these findings in pediatric patients as well [3–5]. Population-based studies have shown that compared to the normal variant homozygosity for this PNPLA3 risk allele is associated with 2 to 4-fold greater risk for NASH and cirrhosis, with up to a 12-fold increased risk for hepatocellular carcinoma and, most recently, an 18-fold increase in liver-related mortality [6, 7]. rs738409 is highly prevalent among those with NASH, with up to 34% of patients homozygous carriers of the mutant alleles [8].

PNPLA3 is a triglyceride (TG) lipase with weak transacylase activity. It is localized to the surface of lipid droplets and is predominately expressed in hepatocytes and stellate cells (HSCs) in human liver [9]. In hepatocytes, PNPLA3 is localized to the surface of lipid droplets and its expression is regulated by pathways involving SREBP1c [10]. PNPLA3 is nutritionally regulated with decreased expression under fasting conditions and increased expression with re-feeding or in the obese [11]. Recently Lindén et al. demonstrated that silencing PNPLA3 in hepatocytes ameliorates NASH and fibrosis in human PNPLA3 I148M knock-in mice but not wild-type mice, supporting a key role of hepatocyte PNPLA3 I148M in disease progression and as a potential target of therapeutic intervention [12].

Less known is the role of PNPLA3 in hepatic stellate cell. HSCs are the primary cell type responsible for extracellular matrix deposition leading to development of hepatic fibrosis in NASH, the only feature associated with liver- and all-cause mortality [13, 14]. PNPLA3 expression is increased following TGF-β-induced activation of HSCs and HSC carrying the I148M PNPLA3 are reported to have a more fibrogenic phenotype [9, 15]. These data suggest that PNPLA3 has a direct role in HSC activation and that the presence of the genetic variant in HSCs potentiates pro-fibrogenic features [9]. In contrast, Pingitore et al. reported that upregulation of wild type PNPLA3 in HSCs results in reduced levels of extracellular proteins involved in fibrosis, suggesting a protective effect of wild type PNPLA3 in HSC [15]. Recently, PNPLA3 expression in NASH biopsies, has been correlated with fibrosis stage and α-SMA levels independently of PNPLA3 genotype [16]. These data, together with a recent report showing an inverse association of PNPLA3 expression with fibrosis stage and collagen 1 mRNA in COL1-positive cells [17] conflict with previous data suggesting a genotype dependent effect on fibrosis and question a direct causal effect of PNPLA3 on the phenotype of the stellate cells. Adding to this uncertainty, another report suggests an indirect role of PNPLA3 in HSCs, where PNPLA3 in hepatocytes may drive activation of HSCs in conditions of high glucose, such as diabetic patients with NASH [16].

To gain a better understanding of the role of PNPLA3 in regulating stellate cell activation, we identified wild-type, heterozygous, and homozygous rs738409 PNPLA3 carriers. HSCs obtained from matched donors were assessed for baseline and TGF-β-induced markers of fibrosis and HSC activation. To directly assess involvement of PNPLA3 in HSC regulation we utilized siRNA targeting human PNPLA3 to downregulate expression.

## Methods

### Primary human stellate cells isolation and culture

Deidentified livers declined for transplantation are used in this study, the patient consent was obtained by www.lifesharing.org. This project (171883XX) has been reviewed by the Director of the UCSD HRPP, IRB Chair, or IRB Chair’s designee and is certified as not qualifying as human subjects research according to the Code of Federal Regulations, Title 45, part 46 and UCSD Standard Operating Policies and Procedures, and therefore does not require IRB review. Livers were graded for steatosis, inflammation, and fibrosis by a pathologist using a double-blinded method [18] and identified as ALD or normal. Primary hHSCs were purified from livers using pronase/collagenase perfusion and gradient centrifugation method, as previously describe [19, 20], cultured for 3 weeks (P0), passaged once (P1) or twice (P2), and analyzed by immunocytochemistry and qRT-PCR. HSC donors were genotyped for the rs738409 SNP as C/C (wild type), C/G (heterozygous), or G/G (homozygous) carriers. Eighty-nine human hepatic stellate cell donors were genotyped as WT (n = 47), heterozygous (n = 31), or homozygous (n = 11) for the rs738409 SNP in PNPLA3. Average donor age (50.6 years), BMI (34.1), and NAFLD activity score (2.14) were similar between all genotypes (Table 1). Passage 2 HSCs were plated in tissue culture treated 6-well plates at a density of 150,000 cells per well in DMEM (Life Technologies, 11965–092) supplemented with 10% FBS (Gemini Bio, 100–106) and 1% Antibiotic Antimycotic solution (Life Technologies, 15240–062) and cultured for 24h. Culture media was exchanged for serum-free starvation media (DMEM + 1% Antibiotic Antimycotic solution). Prior to treatment, primary human HSCs were first fixed by paraformaldehyde then stained with anti-GFAP (Abcam7260 1:100) antibodies, followed by secondary antibody (A-21206, A-32766; ThermoFisher, Waltham, MA). Images were taken using fluorescent microscope (Olympus, Tokyo, Japan), and analyzed using ImageJ software (National Institutes of Health, Bethesda, MD).

**Table 1:** Human hepatic stellate cell donor characteristics.

Donor ID	Age, Gender	BMI	NAFLD Activity Score (NAS)	PNPLA3 Genotype
1	59, M	34	1	C/C
2	56, M	24	0	C/C
3	67, M	37.3	1	C/C
4	24, M	22.9	0	C/G
5	58, F	45.7	0	C/G
6	42, M	32	0	C/G
7	25, F	26	0	G/G
8	41, F	25.6	4	G/G
9	67, M	34.9	0	G/G

### DNA extraction and SNP genotyping

Genomic DNA from the human liver tissues were extracted using QIAamp DNA Kit (Qiagen #51304). Genotyping analysis was performed on Global Screening Array (GSA), which covers over 710,000 SNPs (Illumina) (Diagnomics, San Diego). Briefly, 20ng of DNA was fragmented using the array kit. After the precipitation, DNA was hybridized to microarray followed by washing steps. The hybridized DNA strands on the array were extended, stained and scanned to get the image using iScan (Illumina). The iScan software automatically converted the image to gtc file, which is a compressed binary file containing genotype information. Beeline software (Illumina) was used to generate a human readable genotype table from gtc.

### PNPLA3 silencing and ACC inhibition

For knockdown studies, genotype HSCs were cultured as above followed by transfection with human PNPLA3 siRNA (Ambion) and RNAiMax transfection reagent (Thermo Fisher) prepared in Opti-MEM (Thermo Fisher). The efficiency of siRNA-mediated knockdown was evaluated by real-time PCR. siRNA for PNPLA3 (Ambion Silencer Select s37254) or scrambled negative control (Ambion Silencer Select 4390843) were used at 5nM. After 24 hours of siRNA transfection in serum-free media TGF-β (HumanZyme) was added at 5ng/ml per well, ALK5 small molecule inhibitor (Enzo) was added at 1uM in DMSO as a positive control for HSC inactivation. An ACC inhibitor (CP-640186 –Calbiochem) was dissolved in 100% DMSO for a 10mM stock solution. ACCi stock was diluted to 10uM in HSC media, then added to HSC cultures 30 minutes prior to TGF-β treatment All conditions were controlled for the presence of oligonucleotide and/or DMSO (0.01% final). After 24-hour treatment, cells were collected for gene expression analysis.

### Real-time qPCR gene expression analysis

Total mRNA was extract and purified from human HSCs by RNeasy mini kit plus (Qiagen) per the manufacturer’s instructions. One microgram of RNA was reverse-transcribed to cDNA with SuperScript IV VILO (Thermo Fisher Scientific). and real-time qPCR was performed with TaqMan Mastermix (Thermo Fisher) and TaqMan primer/probes from Thermo Fisher for PNPLA3 (Hs00228747_m1), α-SMA (Hs00426835_g1), COL1A1 (Hs00164004_m1), COL4A1 (Hs00266237_m1), TGF-β (Hs00610320_m1), SREBP-1c (Hs02561944_s1), TIMP1 (Hs01092511_m1), and SMAD7 (Hs00998193_m1). Gene expression was normalized to a house-keeping gene PPIA (Hs04194521_s1) or 18s (Hs03003631_g1) and expressed as fold-change of untreated or control siRNA values.

### Statistical analysis

All data are expressed as mean +/- SD or SEM, as indicated in figure legends. Statistical significance was determined by 1-way or 2-way ANOVA as appropriate, or by using a two-tailed unpaired Student’s *t* test, as indicated in figure legends. P-values <0.05 were considered significant.

## Results

### PNPLA3 I148M does not alter baseline or TGF-β stimulated HSC activation

**Fig 1:**
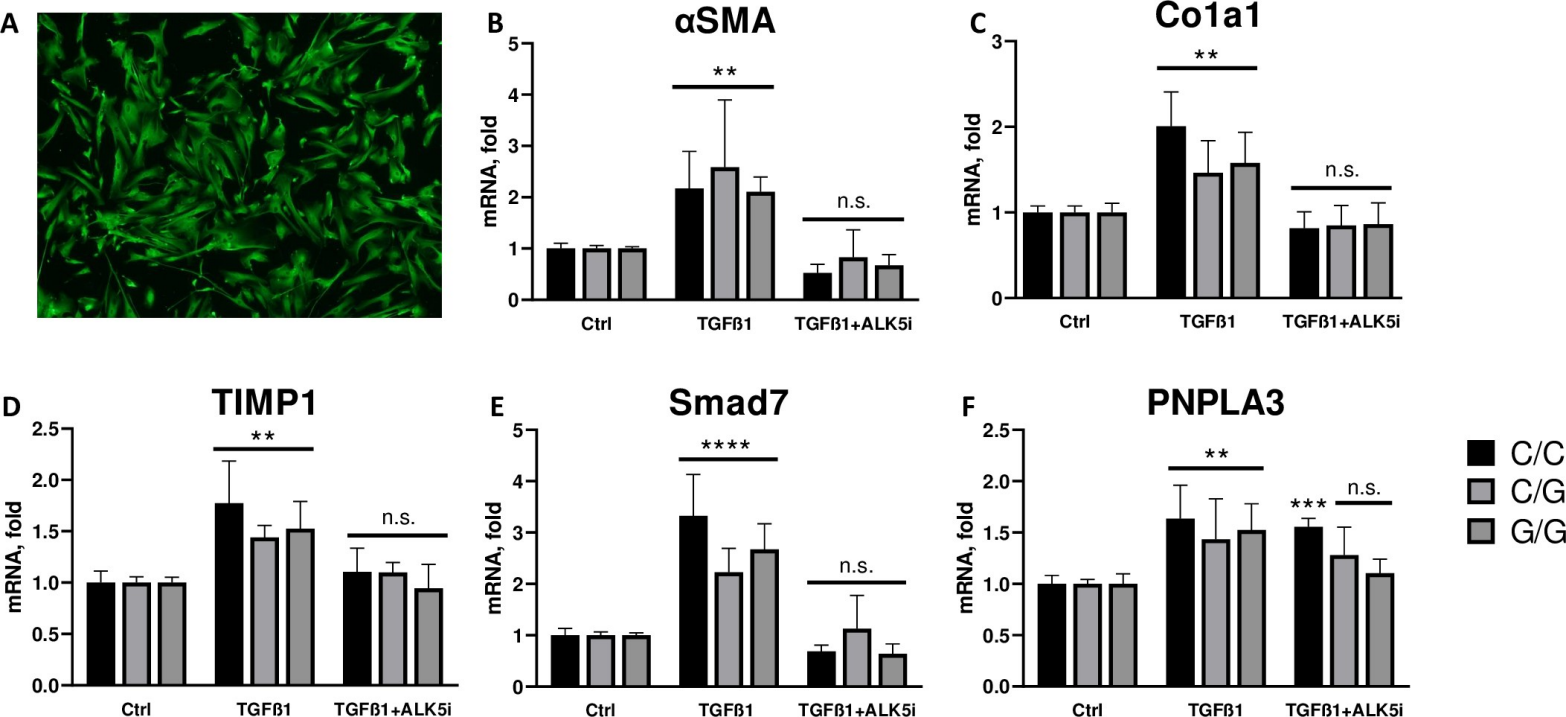
PNPLA3 genotype does not impact activation of HSCs in basal or TGF-β stimulated states. Primary HSCs (n = 20) were genotyped as wild-type, heterozygous, or homozygous for the I148M PNPLA3 mutation. After culture for 24h the cells were imaged and shown to have robust expression of GFAP, an identifying marker of hepatic stellate cells (A). These cells were then treated with or without 5ng/ml TGF-β and mRNA was isolated and qPCR was performed for markers of HSC activation. Baseline activation of HSC was assessed in control cells without TGF-β treatment and activated HSCs were assessed with TGF-β treatment for wildtype (black bar), heterozygous (light grey bar), and homozygous (dark grey bar) donors by expression of α-SMA (B), collagen 1 (C), TIMP1 (D), and SMAD7 (E). Levels of PNPLA3 (F) were measured across genotypes in baseline and TGF-β stimulated conditions. A small molecule ALK5 inhibitor (1mM) was added simultaneously with the TGF-β to serve as a positive control for reversal of activation. Values are mean +/- SD, p = ns within all treatments vs WT genotype by 1-way ANOVA.C/C = wild type PNPLA3; C/G = heterozygous for rs738409; G/G = homozygous for rs738409. n = 6.

Six donors from each genotype were identified for use in subsequent *ex vivo* experiments. Baseline expression of the HSC activation markers of α-SMA, collagen 1, TIMP1, and SMAD7, as well as expression of PNPLA3 were similar between all genotypes (p = ns vs WT genotype for all) (Fig 1A–1E) suggesting that the presence of the I148M mutation in HSCs does not impact their level of baseline activation. TGF-β treatment increased expression of all markers of fibrosis by 1.5 to 3.3-fold across all genotypes after 24h of treatment (p<0.0001 for all vs Ctrl), as well as increased expression of PNPLA3 (~2-fold) equally across all genotypes (p<0.001 vs Ctrl) (Fig 1A–1D; p = ns vs WT genotype for all genes). A small molecule ALK5 inhibitor was included as a positive control for reversal of TGF-β-induced HSC activation and showed similar reductions in markers of fibrosis across genotypes (Fig 1A–1D; p<ns vs Ctrl for all).

### PNPLA3 expression in HSC is not required for an activated fibrotic HSC phenotype

**Fig 2:**
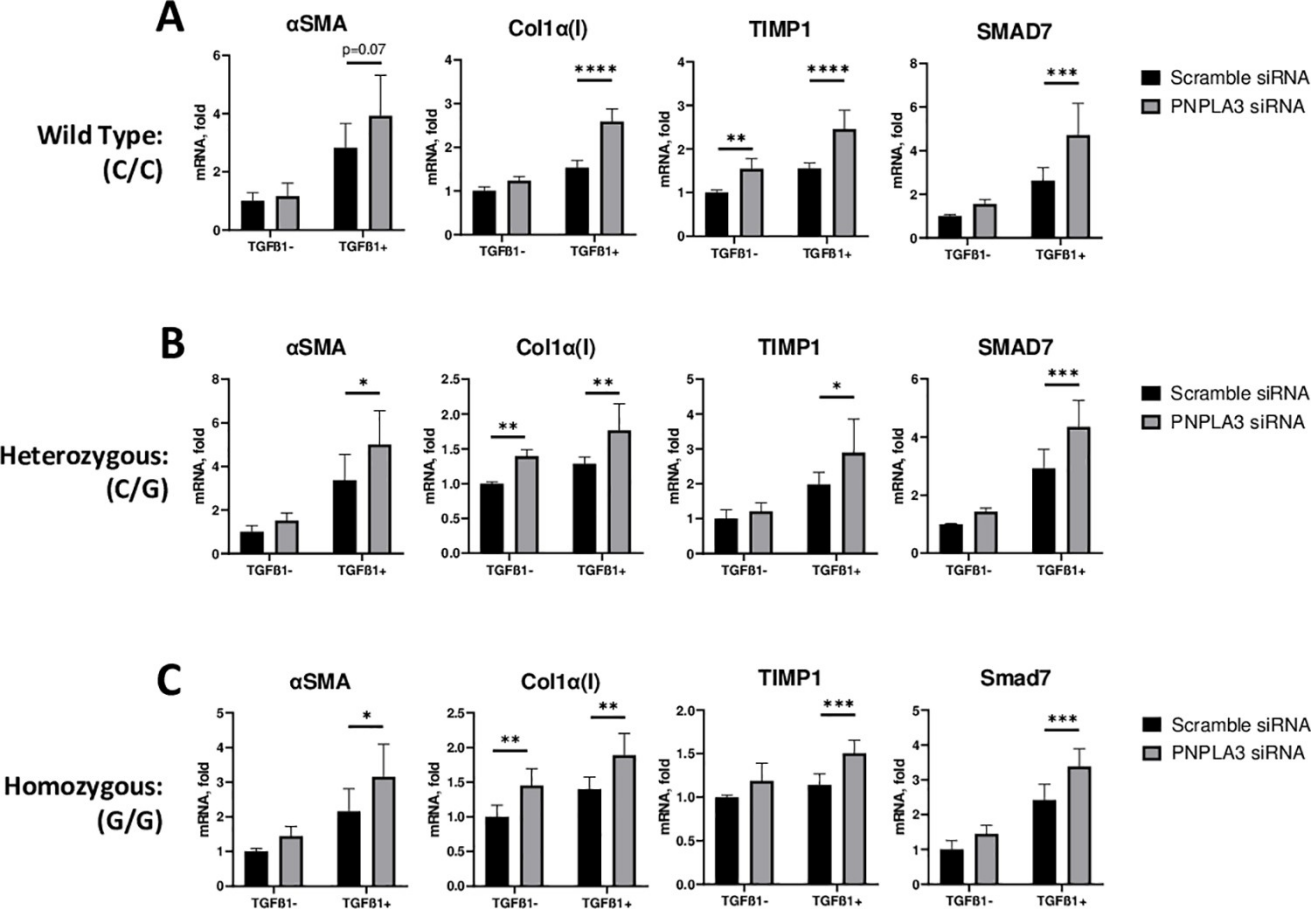
PNPLA3 knockdown activates primary human HSCs regardless of rs738409 genotype. Primary HSCs were treated with control 5nM scrambled or PNPLA3 siRNA (Ambion) followed by treatment with or without 5ng/ml TGF-β and cultured for 24 hours. Expression of α-SMA, collagen 1, TIMP1, and SMAD7 in wildtype (A), heterozygous (B), and homozygous (C) donors was determined by qPCR. Values are mean +/- SD; *p<0.05 **p<0.01 ***p < .001 by 2-way ANOVA vs scramble siRNA. n = 5–6.

Next, to assess if reduction in PNPLA3 expression would impact HSC activation we treated cells with siRNA targeting PNPLA3. siRNA treatment (5 nM) for 48h resulted in PNPLA3 mRNA reduction of 79% on average vs a scramble siRNA control. Knockdown of PNPLA3 had no impact on α-SMA, collagen 1, TIMP1, or SMAD7 expression in the absence of TGF-β (Fig 2A; p = ns vs scramble siRNA for all). In WT PNPLA3 carriers, TGF-β treatment led to increased expression of markers of activation which were further elevated by knockdown of PNPLA3—Col1a1 70%, TIMP1 60%, SMAD7 80% (Fig 2A; *p*<0.05 vs scramble siRNA for all; α-SMA 39% p = ns). For heterozygous and homozygous carriers, treatment with PNPLA3 siRNA similarly increased TGF-β induced HSC activation above that seen with TGF-β alone. Knockdown of PNPLA3 in HSCs carrying either C/G or G/G mutations resulted in increased expression of fibrotic genes in the presence of TGF-β—αSMA 48%, Col1a1 32%, TIMP1 46%, SMAD7 49% for C/G; and 46%, 35%, 32%, 41%, for G/G, respectively (Fig 2B & 2C; p<0.05 vs scramble for all).

### Induction of PNPLA3 in HSCs is associated with decreased activation of fibrotic markers

**Fig 3:**
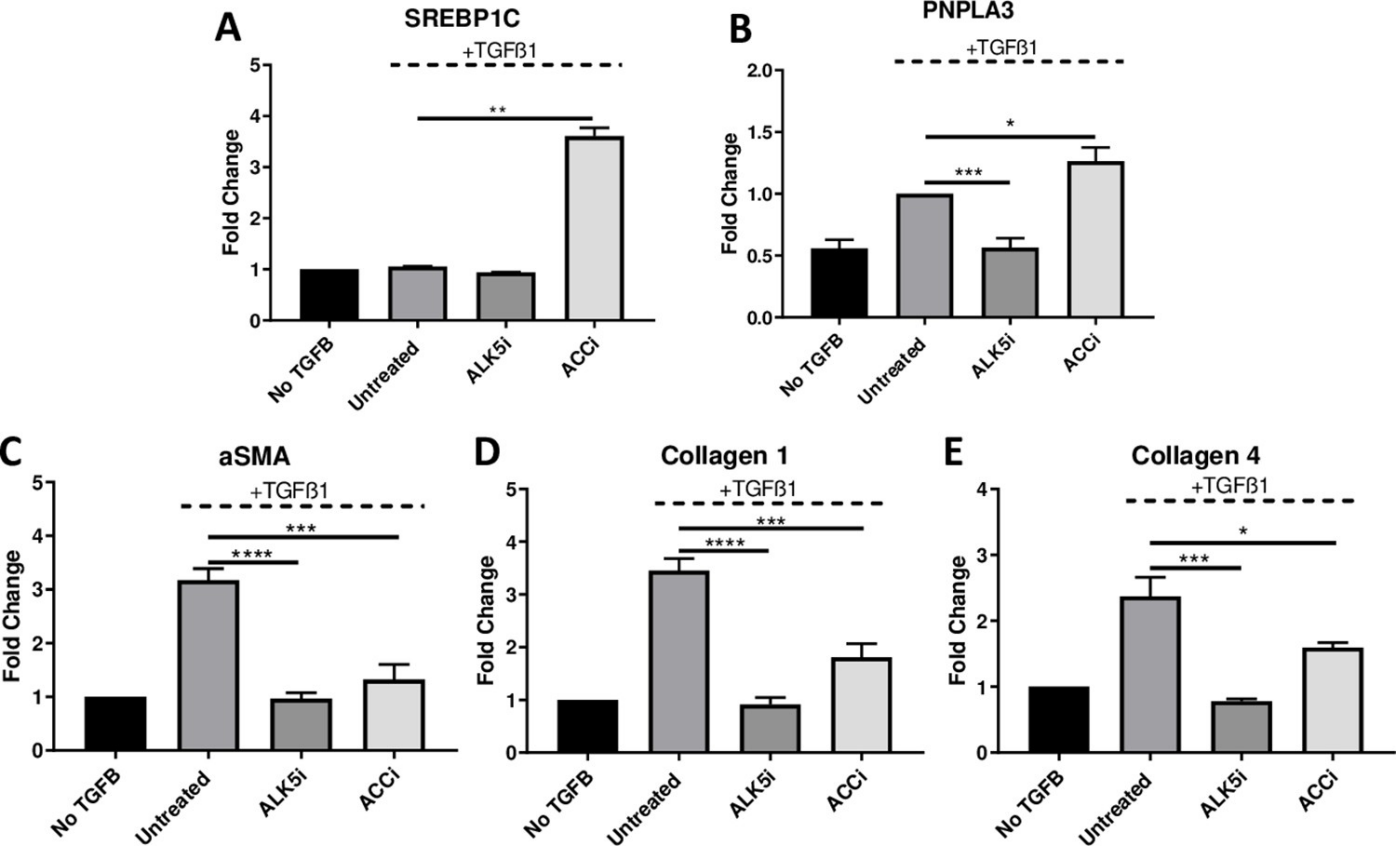
Upregulation of PNPLA3 in primary human HSCs by ACC inhibition. WT PNPLA3 HSCs donors (n = 3) were treated with a small molecule ACC inhibitor at 10uM for 16 hours in the presence or absence of 5 ng/ml TGF-β. A small molecule ALK5 inhibitor (1mM) was added simultaneously with the TGF-β to serve as a positive control for reversal of activation. Transcriptional regulation of SREBP1C (A) and PNPLA3 (B) was demonstrated by qPCR. mRNA expression of HSC activation markers αSMA (C), COL1A1 (D), COL1A4 (E) were also determined by qPCR. Values are mean +/- SEM; *p<0.05 **p<0.01 ***p < .001 by t-test vs untreated condition. n = 2–6.

Acetyl-CoA carboxylase (ACC) is a rate-limiting enzyme in the de novo synthesis of lipids and its inhibition in hepatocytes increases the expression of SREBP-1c, the transcriptional regulator of PNPLA3 [10, 21]. Because ACCi leads to inactivation of stellate cells [22] and upregulation of SREBP-1c in HSCs has been shown to inactivate fibrotic stellate cells [23] we evaluated whether ACC inhibition modulates PNPLA3 in primary human HSC [22, 23]. Human HSCs were treated with ACCi or with a small molecule inhibitor of ALK5 as a positive control in the presence of TGF-β. ACC inhibition increased SREBP-1c expression greater than 3-fold (Fig 3A) and PNPLA3 mRNA was increased by 26% (Fig 3B; p>0.05, vs TGF-β alone). Expression of α-SMA, collagen 1, and collagen 4 were significant reduced following ACC inhibitor treatment by 60%, 51%, and 49% respectively (Fig 3C–3E; p>0.05 vs TGF-β alone). These data suggest that a similar mechanism of SREPB-1c-PNPLA3 regulation is likely present in both hepatocytes and hepatic stellate cells.

## Discussion

The PNPLA3 I148M variant, carried by over a third of NASH patients, is associated with NASH progression, liver cancer, and higher mortality [3–6]. Importantly, downregulation of PNPLA3 only in hepatocytes reduces fibrosis and NASH progression in mice with PNPLA3 I148M knock-in suggesting that PNPLA3 in hepatocytes is responsible for the more aggressive phenotype [12, 24]. Stellate cells have been proposed as an additional site involved in the direct effect of PNPLA3 I148M in NASH [9, 16, 24]. However, the data on PNPLA3 in stellate cells are conflicting with reports of both, protective or pro-fibrotic phenotype [9, 16, 17]. Our data does not support an involvement of HSC PNPLA3 I148M in driving the fibrotic phenotype associated with rs738409 in humans.

While we confirmed that PNPLA3 expression is induced during activation of HSCs [15], our data go on to suggest PNPLA3 induction does not appear to be required for the fibrogenic phenotype of human stellate cells. In our experiments PNPLA3 downregulation resulted in increased expression of profibrogenic factors regardless of the PNPLA3 genotype. In support of this, recent data in humans point to an inverse correlation between PNPLA3 expression and histological scoring of fibrosis, with higher fibrotic stages having lower PNPLA3 expression [17]. Additionally, others reported that increased expression of PNPLA3 in human HSCs resulted in reduced expression of extracellular proteins involved in fibrosis [15]. This last report is consistent with our data showing use of an ACC inhibitor in human HSCs enhanced the expression of PNPLA3 and was associated with inhibition of HSC activation. Together, these data suggest that expression of PNPLA3 in HSCs could be a protective mechanism that restrains activation [23].

A strength of our experimental data is the use of a larger sample size and matched primary human HSCs. Previous experiments have leveraged a limited number of donors and/or utilized the immortalized hepatic stellate cell line LX-2 [25]. These data in LX-2 cells are confounded by the fact that this cell line is a homozygous carrier of the rs738409 SNP [26]. Often, these cells are reported as a “wild-type PNPLA3” HSC phenotype, but as they are actually homozygous mutants these data are difficult to interpret. Additionally, the LX-2 cell line is subjected to trans-differentiation and genetic alterations that further complicate the interpretation of these results [27]. Lastly divergent homology and tissue distribution between rodents and human limited our ability to conduct in vivo studies [9, 28].

In conclusion, our data suggest that PNPLA3 I148M does not play a direct profibrogenic role in HSC during NASH development. These findings have an important clinical implication and suggest that selective downregulation of PNPLA3 mutation in hepatocytes is required to avoid potential deleterious effects of PNPLA3 on HSC activation.

## Supporting information

S1 Dataset (XLSX)
